# An analysis of observer‐rated functional vision in patients implanted with the Argus II Retinal Prosthesis System at three years

**DOI:** 10.1111/cxo.12359

**Published:** 2016-01-24

**Authors:** Duane R Geruschat, Thomas P Richards, Aries Arditi, Lyndon da Cruz, Gislin Dagnelie, Jessy D Dorn, Jacque L Duncan, Allen C Ho, Lisa C Olmos de Koo, José‐Alain Sahel, Paulo E Stanga, Gabriele Thumann, Vizhong Wang, Robert J Greenberg

**Affiliations:** ^1^ Wilmer Eye InstituteJohns Hopkins UniversityBaltimoreMarylandUSA; ^2^ IM3 Inc, Vancouver, Washington, USA; ^3^ Lighthouse Guild InternationalNew YorkNew YorkUSA; ^4^ Moorfields Eye HospitalLondonUnited Kingdom; ^5^ Lions Vision Research and Rehab CenterJohns Hopkins UniversityBaltimoreMarylandUSA; ^6^ Second Sight Medical ProductsSylmarCaliforniaUSA; ^7^ University of California San FranciscoSan FranciscoCaliforniaUSA; ^8^ Wills Eye HospitalPhiladelphiaPennsylvaniaUSA; ^9^ University of Southern CaliforniaLos AngelesCaliforniaUSA; ^10^ Centre Hospitalier National d'Ophtalmologie des Quinze‐VingtsParisFrance; ^11^ Manchester Royal Eye HospitalManchesterUnited Kingdom; ^12^ Hôpitaux Universitaires de GenèveGenevaSwitzerland; ^13^ Retina Foundation of the SouthwestDallasTexasUSA

**Keywords:** Argus II, FLORA, functional vision, retinal prosthesis, retinitis pigmentosa

## Abstract

**Objective:**

The purpose of this analysis was to compare observer‐rated tasks in patients implanted with the Argus II Retinal Prosthesis System, when the device is ON versus OFF.

**Methods:**

The Functional Low‐Vision Observer Rated Assessment (FLORA) instrument was administered to 26 blind patients implanted with the Argus II Retinal Prosthesis System at a mean follow‐up of 36 months. FLORA is a multi‐component instrument that consists in part of observer‐rated assessment of 35 tasks completed with the device ON versus OFF. The ease with which a patient completes a task is scored using a four‐point scale, ranging from easy (score of 1) to impossible (score of 4). The tasks are evaluated individually and organised into four discrete domains, including ‘Visual orientation’, ‘Visual mobility’, ‘Daily life and ‘Interaction with others’.

**Results:**

Twenty‐six patients completed each of the 35 tasks. Overall, 24 out of 35 tasks (69 per cent) were statistically significantly easier to achieve with the device ON versus OFF. In each of the four domains, patients’ performances were significantly better (p < 0.05) with the device ON versus OFF, ranging from 19 to 38 per cent improvement.

**Conclusion:**

Patients with an Argus II Retinal Prosthesis implanted for 18 to 44 months (mean 36 months), demonstrated significantly improved completion of vision‐related tasks with the device ON versus OFF.

## Introduction

The purpose of a retinal prosthetic device is to replace the function of dead or dying photoreceptor cells, which cause visual loss. This is achieved by electrical stimulation of the remaining secondary neurons (for example, ganglion and bipolar cells) in such a way that the signals can be processed by the brain to generate visual perception.

Electrodes intended to stimulate secondary neurons can be implanted on, in or behind the retina, using an epiretinal, subretinal or suprachoroidal approach, respectively.^1^ In the subretinal approach, the implant is placed in the space between the pigment epithelial cells and the dead photoreceptors, whereas in the epiretinal approach, the implant is placed on the surface of the retina. The suprachoroidal electrode array is inserted between the sclera and the choroid.

One of the first clinical applications of retinal prostheses is the Argus II Retinal Prosthesis System (Second Sight Medical Products, Inc, Sylmar, California, USA), which was approved by the Food and Drug Administration (FDA or Agency) in 2013 for treatment of late‐stage retinitis pigmentosa. The device includes a small video camera that is mounted on eyeglass frames. The camera is connected to a processing device worn by the patient, which converts the video input to electronic signals that are transmitted wirelessly to an electrode array (60 electrodes arranged in a 6 × 10 grid) implanted on the surface of the retina (that is, epiretinal implant). The array uses this information to stimulate remaining healthy cells in the retina, and visual information is thereby transmitted by the optic nerve to the brain, where it is perceived as patterns of light. The patient learns to interpret these light patterns.

Safety and benefit of the Argus II System were established from a prospective 30‐patient single‐arm clinical study, which has been previously published.^2^, ^3^ Patients participating in the study had baseline visual acuity of bare light perception or no light perception (that is, 2.9 logMAR in both eyes [worse than 6/4,766 in Snellen notation]), with the device implanted in the worse‐seeing eye. Benefit was established from a suite of assessments, including three computer‐based, objective tests of basic visual skills, intended to measure incremental changes in a profound vision‐loss population. Outcomes demonstrated improvement in visual function, with 33 to 48 per cent of patients scoring better than 2.9 logMAR after three years and one year of use, respectively.

Just as objective tests of basic visual skills had to be developed to establish visual function benefits, standardised assessments are required to evaluate functional vision and quality of life benefits; however, in patients with profound visual loss (defined as 6/150 to 6/300 or logMAR 1.4 to 1.7)^4^ or worse, there are no validated assessments of functional vision. For example, both the National Eye Institute Visual Function Questionnaire (NEI‐VFQ‐25)^5^ and the Veteran Affairs Low Vision Visual Functioning Questionnaire (LV VFQ‐48),^6^ have relatively few items that can be completed by patients with profound vision loss and instead are most appropriate for use with patients with low vision, for example, better than 6/120 or logMAR 1.3.^7^, ^8^

Therefore, the Functional Low‐Vision Observer Rated Assessment (FLORA) instrument was developed specifically for use in patients implanted with retinal prostheses who suffer from profound loss of vision or blindness. The structure and development of FLORA has been previously published.^9^ The instrument combines a self‐report section (Part 1) with a list of functional visual tasks that are rated by a qualified evaluator (Part 2). The tasks were selected based on the requirement that vision be used primarily to achieve some aspect of the task (versus other senses) and that the vision provided by the prosthetic device had the potential to improve performance. For example, selected tasks require the use of light perception (such as, use light from windows to determine orientation), and movement, spatial or form vision (such as, recognise shapes, detect curbs, track another person). Activities requiring higher levels of vision such as reading 12‐point print and driving are not included, as the amount of vision provided by current retinal prostheses is not sufficient to complete these tasks visually. A case‐report narrative (Part 3) summarises the assessor's evaluation of the effect of the Argus II System on the subject's life.

Tasks are organised into four domains, including ‘Visual orientation’, ‘Mobility’, ‘Daily life’ and ‘Interaction with others’. Trained evaluators observed a patient performing each assessed task with the system ON and with it OFF. The ease with which a task is completed is categorised using a four‐point scale.

The purpose of this paper is to analyse observer‐rated functional vision using FLORA (Part 2) in patients implanted with the Argus II System, comparing outcomes with the device ON and OFF.

## Methods

### Study design and patients

Thirty patients were enrolled in a single‐arm, prospective, unmasked clinical trial conducted at 10 centres in the United States and Europe. The study size was limited, reflecting the rarity of the disease, which received a designation of a Humanitarian Use Device from the FDA. Patients served as their own controls, with comparisons made between baseline and post‐implant follow‐up measurements or with the device turned ON and OFF. The trial was conducted in accordance with all relevant national and international regulations for medical device clinical trials including the Declaration of Helsinki. All patients had a minimum of 18 months follow‐up at the time the FLORA was administered. Information on the study design is available from www.clinicaltrials.gov, trial registration number NCT00407602.

Patients were eligible to enrol if they had a confirmed history of retinitis pigmentosa (in the US or Europe) or outer retinal degeneration (in Europe), with bare light perception or worse vision in both eyes, with documentation of functional ganglion cells and intact optic nerve. Exclusion criteria included ophthalmic diseases or conditions that might prevent the Argus II System from working, such as a history of retinal detachments and depression, among other criteria.

## Flora

An assessment of functional visual abilities in and around a residential setting was measured using FLORA. FLORA is a multi‐part instrument primarily developed to obtain an observer‐rated assessment of how patients use a retinal prosthesis in completing a series of common activities of daily living. Thirty‐five tasks are organised into four domains: Visual orientation, Visual mobility, Daily life and Interaction with others. Each of the four domains is intended to measure a different aspect of functional vision. Visual orientation evaluates the ability to use light projection and contrast to improve spatial orientation (that is, understanding where and how one is positioned in the environment). Visual Mobility primarily measures the ability to use vision to detect obstacles. Daily living activities are a series of common activities typically done in a familiar or common environment, such as locating items in a bathroom and locating clothes. Finally, Social interaction measures how patients interact with others in a social setting, including detecting the approach of a person and determining the direction another person is facing.

All assessments were made by qualified evaluators. These evaluators observed a patient performing each assessed task with the system ON and with it OFF (a control condition). The ease with which a task is completed was categorised using a four‐point scale, ranging from easy (score of 1) to impossible (score of 4). In addition, evaluators estimated how much vision is used to accomplish the tasks (ranging from no vision, some vision or only vision).

All FLORA evaluations occurred at the patient's residence and local environment to measure outcomes that were directly relevant to the patients. No effort was made to control lighting (natural or artificial) or other environmental factors to ensure that the assessment was reflective of a ‘real‐world’ experience.

Evaluators were instructed to select tasks based upon each patient's self‐reported goals and activities. As a result, no patient completed all 35 tasks but rather a subset of tasks, which were not necessarily the same from patient to patient.

All evaluators were independent contractors certified in the areas of rehabilitation for the blind, low vision, orientation and mobility or occupational therapy and completed company‐sponsored training on the implementation and use of FLORA. Depending on the centre, one or two therapists implemented the FLORA. When two therapists performed a FLORA for a particular subject, they shared responsibilities; this was usually done with one occupational therapist and one orientation and mobility specialist, who divided the tasks according to their specialties (that is, duplicate assessments were not made). All evaluators were paid for time and out‐of‐pocket expenses. Per the request of the FDA, none of the evaluators were employees of Second Sight or had any financial stake in the Company.

### Statistical analysis

Computations were carried out using SAS 9.4 (SAS Inc, Cary, North Carolina, USA).

Data for all 35 tasks were summarised with the device both ON and OFF, with the difference (ON minus OFF) calculated. The p‐values for paired comparisons were computed using the non‐parametric Wilcoxon signed rank‐test with significance defined as p < 0.05.

Domain values were generated based on the average for all tasks contained within the domain (for example, Orientation is the average of the first six tasks listed in Table 1). The percentage change is defined as the ON mean minus the OFF mean divided by the OFF mean (×100), which is provided for descriptive purposes. Significance for domain values was also calculated using the non‐parametric Wilcoxon signed rank‐test. Adjusted (multiple‐comparison) p‐values for each domain were calculated using the Bonferroni method, which assures that the overall domain Type I error rate (false positive rate) is 0.05 or less.

**Table 1:** FLORA tasks categorised by domain with the population of patients completing each task. A single asterisk (*) indicates tasks that were significantly easier to perform with System ON versus OFF. Double asterisks (**) indicate tasks that were significantly easier to perform with System OFF versus ON. The difference between the mean ON and OFF score is provided, with a negative score representing an improvement in function. Unadjusted (Wilcoxon) p‐values are reported.

Task number	Domain	Task	Number	Mean On minus OFF score	Wilcoxon p‐value
1*	Visual orientation	Locate lights in the environment	26	−1.69	<0.0001
2*	Visual orientation	Find doorways	23	−1.83	<0.0001
3*	Visual orientation	Use light from windows to determine orientation	26	−1.38	<0.0001
4*	Visual orientation	Use artificial light to determine orientation	25	−1.52	<0.0001
5	Visual orientation	Use the sun to determine orientation	10	−0.90	0.0625
6*	Visual orientation	Recognise and use shapes for orientation and environmental information (for example, stop sign)	20	−0.75	0.002
7*	Mobility	Independently cross residential streets by following the lines of a crosswalk	17	−1.00	0.0039
8*	Mobility	Avoid obstacles while walking	24	−0.67	0.0189
9*	Mobility	Estimate the size of an obstacle	22	−0.86	0.0005
10*	Mobility	Avoid low‐hanging branches, plants, head‐high shelves and so on	14	−0.71	0.0078
11*	Mobility	Detect curbs	20	−1.10	0.0002
12*	Daily life	Determine whether room lights are on or off	26	−1.62	<0.0001
13*	Daily life	Locate ordinary objects at various distances (familiar environment)	24	−0.92	0.0105
14*	Daily life	Visually locate a place setting on a dining table	23	−1.30	<0.0001
15*	Daily life	Visually locate/identify things in the bathroom (familiar environment)	11	−0.91	0.0313
16*	Daily life	Visually locate/identify things in the bathroom (unfamiliar environment)	4	−0.25	1.0000
17*	Daily life	Visually locate dishes while washing	11	−1.00	0.0156
18*	Daily life	Visually locate clothes	12	−0.83	0.0156
19*	Daily life	Visually find pots/pans/utensils in the kitchen	13	−0.85	0.0156
20*	Daily life	Sort light from dark laundry	18	−1.89	<0.0001
21 **	Daily life	Travel within home independently	26	0.35	0.0391
22 **	Daily life	Identify top step/bottom step	21	0.76	0.0293
23	Daily life	Negotiate stairways independently	20	0.45	0.1777
24*	Daily life	Cut/chop food	7	0.00	1.0000
25*	Daily life	Identify ordinary objects at various distances	23	−0.83	0.0107
26*	Daily life	Visually identify food on a plate	13	−0.15	1.0000
27	Daily life	Heat/reheat food	7	0.29	0.5000
28	Daily life	Maintain safety: falls/spills/burns	10	0.20	0.5000
29*	Interaction with others	Visually locate people in a non‐crowded setting	26	−1.15	0.0001
30*	Interaction with others	Determine when people walk by	26	−1.23	<0.0001
31*	Interaction with others	Detect the approach of another person	25	−0.88	0.0001
32*	Interaction with others	Determine the direction of movement of people walking by	25	−0.80	0.0010
33*	Interaction with others	Track another person	25	−0.76	0.0005
34	Interaction with others	Visually locate people in a crowded setting	18	−0.33	0.1250
35	Interaction with others	Determine direction another person is facing	22	−0.14	0.5000

## Results

A total of 26 out of 30 eligible Argus II patients were assessed with the FLORA between 18 to 44 months (mean 36 months) after the implant. Data were not available on one patient due to the device being explanted after 14 months. An additional three patients did not consent to the FLORA and thus, were excluded from the analysis.

A list of all 35 tasks, sub‐divided by domain, and the number of patients completing each task (with the device both ON and OFF), is provided in Table 1. Overall, 24 (69 per cent) of the tasks showed a statistically significant improvement in outcome (that is, were easier to perform) with the device ON versus OFF (indicated with an asterisk in Table 1). Two tasks (six per cent) showed a statistically significant decrease in outcome with the device ON versus OFF (indicated with a double asterisk in Table 1). The remainder of tasks (nine or 26 per cent) showed no significant change between ON versus OFF.

For all tasks within the Orientation, Mobility and Interaction domains, there was a significant improvement in task‐completion score with the device ON versus OFF with the exception of tasks 5, 34 and 35 (Table 1: 5. *Use the sun to determine orientation*. 34. *Visually locate people in a crowded setting* and 35. *Determine direction another person is facing*).

Within the 16 items of the Daily Life domain, there was a significant improvement in task‐completion score for all activities with the exception of five tasks: 21 to 23 and 27 and 28. For two of the tasks (21 and 22: *Travel within home independently* and *Identify top step/bottom step*), the OFF data were significantly better than the ON data, whereas for tasks 23, 27 and 28, there was no significant difference between ON and OFF.

The largest improvement in ON versus OFF scores for individual activities was for Tasks 1, 2, 4, 12 and 20 (which ranged from −1.89 to −1.52). All five tasks have in common the ability to use light projection and contrast to identify objects.

The changes in average score of observer‐rated tasks by domain, when the device was ON versus OFF, are provided in Table 2. The ease in which a task is completed is assessed using a four‐point scale, ranging from easy (score of 1) to impossible (score of 4). Thus, a reduction in score reflects an improvement in task completion.

**Table 2:** Change in observer‐rated tasks in patients implanted with the Argus II Retinal Prosthesis System, when the device is ON versus OFF. Scores range from 4 (impossible) to 1 (easy). A negative difference between ON minus OFF represents an improvement in function. Data are summarised by domain. The number of tasks within each domain is provided, as is the average number of patients completing each task (percentage is based on n = 26). SEM is standard error of the mean. Adjusted p‐value is based on the Bonferroni method and is calculated by domain.

Domain	Number of tasks	Mean patients (%)	OFF mean value (SEM)	ON mean value (SEM)	ON minus OFF difference (SEM)	Percentage change	Wilcoxon p‐value	Adjusted p‐value
Orientation	6	22 (85%)	3.56 ± 0.11	2.20 ± 0.17	−1.36 ± 0.19)	−38%	<0.0001	<0.0001
Mobility	5	19 (73%)	3.69 ± 0.10	2.87 ± 0.18	−0.82 ± 0.20	−22%	0.0005	0.0027
Daily life	17	16 (62%)	3.05 ± 0.09	2.47 ± 0.14	−0.58 ± 0.12	−19%	<0.0001	0.0001
Interaction with others	7	24 (92%)	3.92 ± 0.06	3.13 ± 0.16	−0.79 ± 0.15	−20%	<0.0001	<0.0001

As is evident from Table 2, within each domain, ON values were lower (thus better) than OFF values. In each case, the comparison of ON versus OFF was statistically significant. Based on the percent change in score, the Orientation domain showed the largest improvement at 38 per cent with Daily life having the smallest improvement at 19 per cent.

## Discussion

### Assessment design

FLORA was developed to evaluate functional vision of daily life in patients implanted with a retinal prosthesis. During development of the clinical protocol for the Argus II System, the FDA requested that the sponsor evaluate a patient's functional vision in a ‘real‐world’ environment. The Agency noted that ‘laboratory or contrived environments’ might have some use in generating non‐pivotal data but that real world assessments were required for pivotal trials. As a result, the FLORA was developed for use at or near a patient's residence, to use each patient's real‐world environment, including all of the independent variables that would typically be found in a residential setting (glare, shadows, depth, variability in ambient light, variability in weather conditions, et cetera).

FDA also recommended that tasks be selected that pertain to orientation, mobility and activities of daily living. Because blindness is known to be socially isolating,^10^ tasks associated with social interaction were also added to FLORA. The result is a multi‐dimensional instrument intended to assess the effect of restoring some vision to patients with end‐stage disease.

### Potential sources of bias

Because the FLORA measures outcomes with the device both ON and OFF, it provides a self‐control, which helps establish efficacy. This is an important advantage considering that a randomised controlled trial is not practical for a device intended for use in a small population, such as those profoundly blind from a rare disease like retinitis pigmentosa; however, neither the evaluator nor the patient is masked as to the operational status of the device. The nature of the prosthesis requires extensive head movements to scan across a visual scene, which eliminates the possibility of masking for the evaluator. Furthermore, the device provides auditory signals that are crucial to its operation, which makes masking for the patient difficult.

A control condition using settings with a ‘scrambled’ spatial map, which has been used with Argus I and Argus II subjects in the past,^11^, ^12^ could be implemented and subjects could be masked to this condition; however, it would be logistically challenging. The scrambled spatial map would have to be created and uploaded in the clinic, by site staff or Second Sight personnel. As the FLORA is done at the subject's home, by rehabilitation professionals, there would be logistical challenges. Moreover, the purpose of a scrambled spatial map control is to determine whether a particular task requires spatial vision (in this context, whether subjects can determine relative positions of different electrodes and make use of spatial information in the visual scene). While this is a scientifically interesting question, it has little direct relevance to subjects’ lives. The true determination of whether the Argus II System is useful to patients in the real world is whether they can perform functional tasks better or more easily with the System than they can without it – that is, System ON or OFF.

Although masking was not attempted, other means of reducing bias were incorporated in the FLORA study.^13^ As suggested by the FDA, all evaluators were independent of the sponsor and clinical team, having extensive training and experience working with patients with profound visual loss. The test and all tasks were clearly explained, eliminating ambiguity as to the nature of the task and how outcomes should be measured. Tasks were generally completed in a prescribed order with the device first in the ON position and then the OFF position. This ensured that familiarity with the task could bias outcomes in favour of the OFF position, since the same task was first completed with the device ON.

At the request of FDA, evaluators pre‐selected tasks, based on a patient's goals and routine activities, which may be perceived as a source of potential bias. This resulted in as few as four patients completing one task within the Daily life domain, although participation rates averaged 19 patients per task overall (73 per cent) and were much higher within the other domains (for example, 24 or 92 per cent of patients on average for tasks within the Interaction‐with‐others domain; 22 or 85 per cent of patients on average for tasks within the Orientation domain).

On average, testing was done 36 months after the device was implanted. Although it is generally accepted that devices have a tendency to invoke a larger placebo response than pharmaceuticals,^14^ the long follow‐up period had the potential to mitigate any placebo response on the part of the patient. All patients had considerable experience with the device before testing was completed, and had learned how and where to use the device to have the best outcome.

### Findings

Results from the FLORA Part 2 (observer‐rated tasks) establish that for all four domains, most of the tasks were significantly easier to perform with the system ON versus OFF. In particular, tasks related to the use of light projection and contrast to detect objects (that is, primarily categorised within in the Orientation domain) had the largest difference in favour of the device being ON. This suggests that Argus II provides its greatest advantage in environments with maximum light contrast (that is, involving directed light sources, such as an overhead light).

With respect to the two tasks where Argus II was not effective (that is, OFF data were statistically better than ON data), both tasks were within the Daily life domain (tasks 21 and 22) and involved mobility within the home. This outcome is not surprising considering that within a residential environment, which is both controlled and familiar, blind patients develop a travel routine that is based more on memory than on vision. Nonetheless, the overall Daily living domain remained highly significant in favour of using Argus II.

Travel outside of a controlled environment is measured with the Mobility domain, where it was found that Argus II significantly improved task completion. The ability to safely manoeuvre across streets and on sidewalks, while avoiding obstacles is an important advantage of Argus II. This in turn could mitigate the risk of falls and other injuries, which are higher in patients with visual impairment.^15^

Finally, the Interaction domain is also highly significant in favour of using Argus II. Tasks within the domain measure the detection, tracking and observation of people in a social setting, which is important in the development of relationships and a strong social network.

Overall outcomes reported from analysis of the FLORA Part 2 are particularly noteworthy when considering that all subjects in the Argus II study experienced end‐stage retinitis pigmentosa with profound visual loss. Patients frequently had a long history of blindness, with virtually no hope of restoration of vision. Although subjects achieved only modest improvement in visual acuity from use of the device (as measured on the logMAR scale), functional visual tasks were significantly easier to perform with the System than without it, as measured by the FLORA observer‐rated tasks. This point is consistent with the general shift in medicine away from surrogate outcome measures that may be easily quantified, toward outcomes that are directly relevant to patients, such as functional vision.

## Conclusion

The FLORA was developed in accordance with FDA guidance to measure functional vision in a patient's own living and working environment. Part 2 of the instrument was used to measure the change in observer‐rated tasks in patients implanted with the Argus II Retinal Prosthesis System, when the device was ON versus OFF. In all domains and the majority of individual tasks, ON data were significantly better than OFF data, meaning that task completion was easier when patients used the Argus II System. Improvements ranged between 19 to 38 per cent within the four domains, with the greatest improvement measured in tasks involving light projection and contrast. These data demonstrate that within a population with profound visual loss treated with a retinal prosthesis, measuring functional vision is relevant to establishing efficacy and utility.

## Disclosures

Dr Jessy Dorn and Dr Robert Greenberg are employees and stockholders of Second Sight Medical Products.
